# Vestibular Functions and Parkinson's Disease

**DOI:** 10.3389/fneur.2018.01085

**Published:** 2018-12-11

**Authors:** Paul F. Smith

**Affiliations:** ^1^Department of Pharmacology and Toxicology, School of Biomedical Sciences and The Brain Health Research Centre, University of Otago, Dunedin, New Zealand; ^2^Brain Research New Zealand Centre of Research Excellence, Eisdell Moore Centre for Hearing and Balance Research, University of Auckland, Auckland, New Zealand

**Keywords:** vestibular system, Parkinson's disease, vestibulo-ocular reflexes, vestibulo-spinal reflexes, VEMPs, striatum, dopamine

## Abstract

For decades it has been speculated that Parkinson's Disease (PD) is associated with dysfunction of the vestibular system, especially given that postural instability is one of the major symptoms of the disorder. Nonetheless, clear evidence of such a connection has been slow to emerge. There are still relatively few studies of the vestibulo-ocular reflexes (VORs) in PD. However, substantial evidence of vestibulo-spinal reflex deficits, in the form of abnormal vestibular-evoked myogenic potentials (VEMPs), now exists. The evidence for abnormalities in the subjective visual vertical is less consistent. However, some studies suggest that the integration of visual and vestibular information may be abnormal in PD. In the last few years, a number of studies have been published which demonstrate that the neuropathology associated with PD, such as Lewy bodies, is present in the central vestibular system. Increasingly, stochastic or noisy galvanic vestibular stimulation (nGVS) is being investigated as a potential treatment for PD, and a number of studies have presented evidence in support of this idea. The aim of this review is to summarize and critically evaluate the human and animal evidence relating to the connection between the vestibular system and PD.

## Introduction

Parkinson's Disease (PD) is a chronic neurodegenerative disease characterized by tremor, rigidity, slowness of movement (“bradykinesia”), postural imbalance, and, ultimately, other non-motor symptoms such as cognitive impairment and depression (1). The age- and gender-adjusted incidence rate of PD is approximately 13.4 per 100,000, which rapidly increases over the age of 60 (2, 3). The fact that postural instability is a symptom of the disease, suggests the possibility that the vestibular system may be implicated. Nonetheless, conclusive evidence for vestibular dysfunction in PD has been slow to emerge.

The notion that vestibular dysfunction may occur in PD has a long and complicated history. Studies reaching back into the 1980's have suggested such a link, but several studies have reported negative results [e.g., (4); see (5) for a review]. Similarly, there has been a suggestion that vestibular information is transmitted to the basal ganglia, the striatum in particular, which loses dopaminergic input in PD. However, confirmation of such a pathway has also been slow to emerge, with apparent discrepancies between various electrophysiological and neurotracer studies [see (6) for a review]. Over the last 10 years, interest in the effects of vestibular stimulation on the basal ganglia has been amplified by studies reporting that noisy galvanic vestibular stimulation [nGVS; e.g., (7)] or caloric vestibular stimulation [e.g., (8)] can reduce the severity of some PD symptoms (see (5) for a review). From these studies it has been suggested that some form of vestibular stimulation may be a potential early adjunctive treatment for PD, that may delay the need for drug treatments such as L-DOPA and ropinirole, or at least reduce the doses needed so that higher doses of drug therapy can be “saved” for later in the course of the disease.

The objective of this review is to summarize and critically evaluate the current evidence for an interaction between vestibular function and PD, considering: (1) the evidence that vestibular symptoms are present in PD; (2) whether there is evidence for the neuropathology of PD in the central vestibular system; (3) what neural circuitry might underlie an interaction between the vestibular system and the striatum; and (4) whether vestibular stimulation can affect the severity of PD symptoms.

## Vestibular Symptoms in Parkinson's Disease

Many early studies of vestibular function in PD reported evidence of deficits in the vestibulo-ocular (VORs) and vestibulo-spinal reflexes (see Tables 1, 2). There seem to have been relatively few VOR studies reported (Table 1); however, it is conceivable that deficits could appear in the vestibulo-spinal reflexes without necessarily being evident in the VORs or the perception of vertical, since the VORs and vestibulo-spinal reflexes involve relatively independent neural pathways (21). The technology for detecting vestibular deficits of various sorts has advanced enormously in recent decades and it is conceivable that in some early studies, vestibular dysfunction was present but was not detected. Of course, one of the critical factors is the stage of the PD, with vestibular symptoms perhaps more likely to be detected later in the disease. Furthermore, in some studies, PD patients exhibiting vestibular symptoms may have been excluded from the study.

**Table 1:** A summary of studies examining nystagmus and VOR function in PD patients.

Reichert et al. (9)	36 PD patients	Reduced or absent caloric nystagmus
	316 controls	
Ciparrone et al. (10)	36 PD patients	Abnormal caloric nystagmus
	316 controls	
Vitale et al. (11)	11 PD patients	Unilateral vestibular hypofunction and positional and SN in patients with LTF
	11 controls	
Lv et al. (12)	63 PD patients	Abnormally high VOR gain
	56 controls	

**Table 2:** A summary of studies examining posture and VEMPs in PD patients.

Pastor et al. (4)	15 PD patients	No difference in body sway
	10 controls	
Pollak et al. (13)	54 PD patients	Unilaterally absent cVEMPs 37% and unilaterally absent cVEMPs 7.4% of patients
	53 controls	
Potter-Nerger et al. (14)	20 PD patients	Smaller cVEMPs in patients L-DOPA increased cVEMP Amplitude
	10 controls	
De Natale et al. (15)	14 early PD patients	Delayed cVEMPs, mVEMPs and oVEMPs
	19 advanced PD	Absent VEMPs
	27 controls	
De Natale et al. (16)	24 PD patients	Abnormal cVEMPs, mVEMPs and oVEMPs in PD patients
	24 controls	
Potter-Nerger et al. (17)	13 PD patients	cVEMPS preserved in patients
	13 controls	oVEMPS significant delay and reduced amplitude in patients
Venhovens et al. (18)	30 PD patients	Delayed CVEMPs and oVEMPs in PD patients
	14 Atypical P	
	25 controls	
Shalash et al. (19)	15 PD patients	Absent oVEMPs and delayed cVEMPs in patients
	15 controls	
Huh et al. (20)	25 FOG PD patients	Diminished sensory processing in in PD patients with FOG
	22 no FOG PD	
	26 controls	

### Vestibulo-Ocular Reflex Studies

PD is well known to be associated with deficits such as hypometric saccades and abnormalities of smooth pursuit eye movement (e.g., see (22) for a review). Whether the VORs are affected is still somewhat controversial, even decades after the first studies.

Reichert et al. (9) studied bi-thermal caloric nystagmus in 36 PD patients and 316 controls and found reduced or absent nystagmus in the patients, which was associated with postural instability (Table 1). Ciparrone et al. (10) studied the effects of caloric-induced nystagmus in 36 PD patients and 316 controls and found abnormal nystagmus in 82.9% of the patients. However, they observed few cases of spontaneous nystagmus. In general, caloric stimulation generated an increased response in many cases (48.6%), sometimes with a directional preponderance (25.7%). Nonetheless, the abnormal nystagmus was not correlated with the clinical PD symptoms. The results for the caloric nystagmus did not appear to be analyzed statistically. Vitale et al. (11) studied vestibular function using caloric testing and video-oculography in 11 PD patients and 11 age-matched controls. They found evidence of unilateral vestibular hypofunction in all of the patients with lateral trunk flexion, a common symptom in PD. They observed spontaneous positional jerk nystagmus with primary forward gaze, which was suppressed by visual fixation, as well as positional nystagmus. Nystagmus was increased during the head shaking test in all patients except one. The results were analyzed statistically (see Table 1).

Lv et al. (12) are one of the few groups to quantitatively report abnormalities in the head impulse test in PD patients. They used the video head impulse test in 63 PD patients and 56 controls. They found, somewhat paradoxically, that the VOR gain for PD patients was significantly greater than the controls (i.e., 1.2 and 1.23 for the left and right sides compared to 0.98 and 0.99 for the control group, respectively). However, this result may be consistent with the increased caloric nystagmus reported by Cipparrone et al. (11). Lv et al. (12) found no correlation between the VOR gain and age or disease duration and only a weak correlation between the VOR gain and the Unified Parkinson's Disease Rating Scale score. The authors speculated that the increased VOR gain was possibly a compensatory response which developed during the early stages of PD.

Taken together, these studies suggest that there may be VOR abnormalities in PD; however, as yet too few quantitative studies have been conducted to draw reliable conclusions.

### Vestibulo-Spinal Reflex Studies

Pastor et al. (4) examined the postural response to galvanic vestibular stimulation (GVS) while standing with feet together and eyes closed, in 15 PD patients and 10 age-matched controls. They observed no significant difference in the speed or direction of body sway between the patients and controls, suggesting that a central vestibular dysfunction was not responsible for their postural instability.

Pollak et al. (13) examined the cervical vestibular-evoked myogenic potentials (cVEMPs), indicative of saccular function, in 54 PD patients and 53 controls and found unilaterally absent cVEMPs in 37% of the patients and bilaterally absent cVEMPs in 7.4%, which were statistically significant differences compared to the controls. Once again, however, there was no correlation with the disease stage. Potter-Nerger et al. (14) also studied cVEMPs in 20 PD patients (10 with and 10 without sub-thalamic electrodes) and 10 age-matched controls. They observed significantly smaller cVEMPs in the PD patients, but especially in those without the sub-thalamic electrodes. They found that administration of L-DOPA, but not sub-thalamic stimulation, increased the cVEMP amplitude.

de Natale et al. (15) studied cervical, masseter and ocular VEMPs (cVEMPs, mVEMPs, and oVEMPs) in 14 patients with early PD, 19 with advanced PD and 27 age-matched controls, and found that the VEMPs were abnormal in the PD patients, although these were different in the early and advanced patients. The mVEMP and oVEMP amplitudes were significantly smaller than controls for the late PD group and the frequency of abnormalities for each VEMP was significantly higher than controls. PD is commonly associated with sleep disorders (see (23) for a review) and the severity of this problem is often quantified using the REM Sleep Behavior Disorder Screening Questionnaire (see (24) for a review). In this study the degree of VEMP impairment was found to correlate with the REM Sleep Behavior Disorder Screening Questionnaire in both groups of PD patients and inversely with the Mini-BEST test scores (measuring postural instability) in the advanced PD patients. In a further study (16), they investigated cVEMPs, mVEMPs and oVEMPs in 24 PD patients and 24 age-matched controls and found that cVEMPS were abnormal in 41.7% of PD patients, mVEMPS in 66.7% and oVEMPs in 45.8%. For mVEMPs and oVEMPs, but not cVEMPs, the amplitudes were significantly smaller than the control group. There was also a significant correlation and a significant inverse correlation between the number of abnormal VEMPs and the scores on the REM Sleep Behavior Disorder Screening Questionnaire and the mini-BEST test, respectively. These results again suggest the possibility that VEMPs might be useful in the diagnosis of PD (see Table 2).

Potter-Nerger et al. (17) studied cVEMPs and oVEMPs in 13 PD patients and 13 age-matched controls. They found that the cVEMPS were relatively well-preserved in the PD patients; however, the oVEMPs, indicative of utricular function, exhibited a significant delay and a significantly reduced amplitude. Furthermore, L-DOPA treatment had no significant effect on either the cVEMPs or the oVEMPs.

Venhovens et al. (18) examined cVEMPs, oVEMPs and brainstem auditory-evoked potentials in 30 PD patients, 14 with Atypical Parkinsonism and 25 age- and sex-matched controls. In addition, they measured the subjective visual vertical and used videonystagmography with caloric and rotatory chair stimulation. They found that 27 of the 30 PD patients and all 14 Atypical Parkinsonism patients had significantly abnormal cVEMPs and oVEMPs, compared to the controls. In PD and Atypical Parkinsonism patients, brainstem auditory-evoked potentials exhibited a significant delay. Delayed latencies for oVEMPs and cVEMPs were common for the PD and Atypical Parkinsonism groups. The abnormal vestibular test results were correlated with an increased risk of falling. Once again, these results support the hypothesis that the symptomatology of PD includes vestibular dysfunction.

Shalash et al. (19) studied the relationship between oVEMPs, cVEMPs and brainstem auditory-evoked potentials in 15 patients with PD and 15 age-matched controls. They found that the PD patients exhibited significantly delayed brainstem auditory-evoked potential latencies as well as absent and delayed oVEMPs and delayed latencies for cVEMPs. The ipsilateral and contralateral cVEMPs were significantly correlated with measurements of sleep, perception, memory and cognition, as well as urinary scores. The VEMP responses were significantly correlated with cardiovascular function and sexual dysfunction.

Huh et al. (20) used the “sensory organization test” to study vestibular contributions to postural control in 25 PD patients with freezing of gait, 22 PD patients without freezing of gait and 26 age-matched controls. The sensory organization test comprises 6 conditions in which postural stability is challenged by changing visual and somatosensory input, thereby altering the dependence on vestibular input (20): (1) eyes open, floor fixed, visual surround fixed; (2) eyes closed, floor fixed, visual surround fixed; (3) eyes open, floor fixed, visual surround sway-referenced; (4) eyes open, floor sway-referenced, visual surround fixed; (5) eyes closed, floor sway-referenced, visual surround fixed; (6) eyes open, floor sway-referenced, visual surround sway-referenced. They found that the PD patients with freezing of gait exhibited significantly worse postural sensory processing, especially the inability to use vestibular information.

Taken together, these studies suggest an emerging consensus that VEMPs become abnormal in PD.

### Subjective Visual Vertical and Perception of Tilt Studies

Bronstein et al. (25) evaluated the ability to set a straight line to gravitational vertical (“subjective visual vertical”) in 24 PD patients, 8 patients with bilateral vestibular loss and 24 control subjects. They used static conditions as well as changes in body position and background visual motion. They found no statistically significant differences in the subjective visual vertical in the upright position. However, while the subjective visual vertical was significantly different during visual motion for the patients with bilateral vestibular loss, the PD subjects performed similarly to the control subjects. However, Scocco et al. (26) studied 8 PD patients, 9 patients with “Pisa Syndrome,” a condition in which a person exhibits a lateral deviation around the longitudinal axis for no obvious reason, and 18 controls. They tested the subjective visual vertical when the PD patients were on or off L-DOPA and found that the PD patients performed significantly worse than the controls when they were on the L-DOPA, and visual dependency was greater for the PD patients when they were inclined, during the off condition. Barnett-Cowan et al. (27) studied 12 PD patients and compared them with 13 age-matched controls and found that PD patients with left-sided initial motor symptoms, were more dependent on visual information for the subjective visual vertical, when they were taking their dopaminergic medication.

More recently, Bertolini et al. (28) examined the judgement of forward tilt in 11 PD patients and compared it to 19 age-matched controls. This was done on a motion platform in darkness in response to two consecutive forward tilt movements, and combining tilt movements with translations in order to probe multi-cue integration. They found that PD patients were significantly less accurate in judging forward tilt, but only in the multi-cue conditions, not the single cue conditions, suggesting a deficit in sensory integration.

Finally, in the most recent study published, Gandor et al. (29) studied subjective visual vertical in 30 patients with and without lateral trunk flexion and found that PD patients with lateral trunk flexion had significantly greater subjective visual vertical angles than those without lateral trunk flexion. Fourteen out of 21 patients with lateral trunk flexion exhibited abnormal subjective visual vertical while 9 out of 9 patients without lateral trunk flexion exhibited a normal subjective visual vertical.

Taken together, these studies suggest that if subjective visual vertical is abnormal in PD, it may be related to whether the patients are taking L-DOPA and whether they suffer from lateral trunk flexion. Too few studies of forward tilt have been conducted to draw reliable conclusions.

### Other Studies

Montgomery et al. (30) studied orientation to a starting position in 48 PD patients (24 with mild disease and 24 with moderate disease) and 35 control subjects, passively transported in a wheelchair in a visual condition where they could see the walls and ceiling but not the floor, and a “vestibular” condition, in which they wore a blindfold. They found that the PD patients with moderate disease performed significantly worse in the visual and vestibular conditions compared to the control subjects and patients with mild PD, but that while performance in the visual condition distinguished the mild PD patients from the controls, the mild PD patients and the controls performed similarly in the vestibular condition compared to the moderate PD patients.

Putcha et al. (31) used fMRI to study cortical activation in areas involved in processing visual motion, in 23 PD patients and 17 matched controls. They examined V6, V3a and the medial temporal area, as well as two regions associated with visual-vestibular processing, the parieto-insular vestibular cortex and the cingulate sulcus visual area, stimulated with simulated optic flow motion as well as random motion. Compared to the control subjects, the PD patients exhibited significantly reduced activity in the medial temporal area and cingulate sulcus visual areas, and activation of the cingulate sulcus visual area was inversely correlated with the disease severity.

It has been suggested that the cortical field potential responses (“electrovestibulography”) to vestibular stimulation might be used as a biomarker for the diagnosis of PD (32–34). Electrovestibulography is a technique in which the shape and phase of field potential signals in response to natural vestibular stimulation (e.g., tilting), are analyzed using algorithms such as the Neural Event Extraction Routine (NEER) (32–34). Classification statistical analyses such as linear discriminant analysis are then used in an attempt to classify or diagnose patients as having PD, based on the field potential data, and the results are interpreted using receiver-operating characteristic (ROC) curves, in terms of the sensitivity [i.e., true positive/(true positive + false positive)] and specificity [i.e., true negative/(true negative + false positive)] of the diagnosis. Dastgheib et al. (32–34) have described high levels of diagnostic accuracy (up to 95%) using multivariate statistical and data mining methods such as linear discriminant analysis. The same method has been used to demonstrate that treatment of PD with L-DOPA may disturb vestibular function (35, 36); see also (37).

Hwang et al. (38) conducted an interesting study of 8 PD patients in which they stood in a visual cave with optokinetic stimulation at 0.2 Hz while simultaneously receiving an 80 Hz vibratory stimulus to their Achilles tendons and a bilateral monopolar GVS stimulus at 0.36 Hz. The amplitude of the visual stimulus was varied so that the weighting of vision changed, and the gain of the proprioceptive and vestibular stimuli was also varied. In humans without PD, they found that increasing the amplitude of the visual input caused them to reduce the emphasis on visual input, as well as re-weighting visual-proprioceptive and visual-vestibular interaction effects, suggesting that they used intermodal re-weighting to adapt to the situation. By contrast, they found that PD patients had difficulty with cross-modal interaction, suggesting that they suffered from a deficit in fusing information from different sensory modalities.

Taken together, these studies suggest that PD patients may experience deficits in the way that the brain integrates sensory information, including vestibular information, and that these changes, in terms of electrophysiological activity, could potentially form a signature of PD which might be useful in early diagnosis of the disease.

## Parkinson's Disease Pathology in the Central Vestibular System

Seidel et al. (39) conducted a pathological study of α-synuclein, which forms Lewy bodies, in the hindbrains of 5 PD patients, 1 patient with Parkinson's Disease with dementia and 5 with dementia with Lewy bodies. In all cases they found Lewy bodies and Lewy neurites in the substantia nigra, ventral tegmental area, pedunculopontine tegmental nucleus, raphe nuclei, periaqueductal gray, locus coeruleus, parabrachial nuclei, reticular formation, dorsal motor vagal and solitary nuclei, in addition to the vestibular nucleus complex, prepositus hypoglossi, and even the root of the vestibular nerve. The subnuclei of the vestibular nucleus complex included the medial vestibular nucleus, superior vestibular nucleus and the lateral vestibular nucleus. These results suggest very strongly that the neuropathology of PD extends into the central vestibular system and is therefore likely to undermine at least some of the vestibular reflexes, as well as autonomic, limbic system and cortical projections carrying vestibular information.

Muller et al. (40) studied cholinergic terminals in the thalamus and cortex, and dopamine (DA) terminals in the striatum, in 32 males and 92 females with PD, and 10 female and 15 male age-matched controls. They used positron emission tomography (PET) for the vesicular monoaminergic transporter to image DA terminals and acetylcholinesterase for the acetylcholine terminals and related these data to data from the sensory organization test balance platform protocol. They found that reduced cholinergic thalamic innervation was related to increased center of pressure sway speed, while controlling for the effects of Parkinsonian motor deficits and cognitive impairment. However, there were no significant effects of cortical cholinergic terminal deficits or striatal DA terminal deficits. The authors suggested that PD symptomatology is modulated by connections between the pedunculopontine tegmental nucleus and the thalamus. This is very interesting since the pedunculopontine tegmental nucleus may be one of the nuclei through which vestibular information reaches the dorsal striatum (see (6) for a review). The pedunculopontine tegmental nucleus, which contains vestibular-responsive neurons (41), also undergoes significant changes in the number of cholinergic neurons following bilateral vestibular loss in rats (42). Cai et al. (43) have recently used fMRI to examine the connectivity between the pedunculopontine tegmental nucleus and other brain regions following GVS. They used 23 PD patients without evidence of freezing of gait and who were on medication (L-DOPA) and compared them with 12 controls. They reported that GVS did not have a significant effect on pedunculopontine tegmental nucleus connectivity in controls; however, in PD patients, while the baseline magnitude of pedunculopontine tegmental nucleus connectivity was inversely correlated with Unified Parkinson's Disease Rating Scale scores, both noisy and sinusoidal GVS elevated the level of pedunculopontine tegmental nucleus connectivity, increasing it with respect to the inferior parietal region. They found that noisy GVS reduced its connectivity with the basal ganglia and cerebellum. This appears to be the first study to demonstrate that GVS can modulate brain connectivity in patients with PD and therefore the results are highly relevant to those studies, reviewed later, that have used noisy or stochastic GVS in an attempt to reduce Parkinsonian symptoms.

Wellings et al. (44) recently studied the expression of non-phosphorylated neurofilament protein in the lateral vestibular nucleus (i.e., Deiters' nucleus), which are proteins whose reduced expression in the substantia nigra is known to contribute to impaired motor function. They conducted immunohistochemical analysis of the brainstems of 6 PD patients and 6 aged-matched controls and found that there was a 50% reduction in the expression of non-phosphorylated neurofilament protein in the lateral vestibular nucleus; by comparison, there was no significant difference in the facial nucleus, demonstrating that this effect was selective for the lateral vestibular nucleus. There was a similar decrease in the intensity of non-phosphorylated neurofilament protein labeling in the lateral vestibular nucleus of PD patients. They also reported an 84% increase in somatic lipofuscin in the lateral vestibular nucleus of PD patients, the significance of which is that lipofuscin deposits are known to increase with neurodegeneration. The authors suggested that these changes in the lateral vestibular nucleus are probably related to the postural deficits seen in PD.

Taken together, these studies provide substantial evidence of neuropathological changes in the vestibular nucleus complex during the development of PD. In addition to explaining some of the abnormalities in vestibular reflexes, especially VEMPs, that have been reported in PD patients, this may also explain some of the cognitive deficits that eventually develop in PD, due to deterioration of the ascending pathways from the vestibular nucleus complex to the limbic system and neocortex [see (45, 46) for a review].

## Neural Circuitry Underlying Effects of Vestibular Stimulation on Parkinsonian Symptoms

### Animal Studies

**Figure 1:**
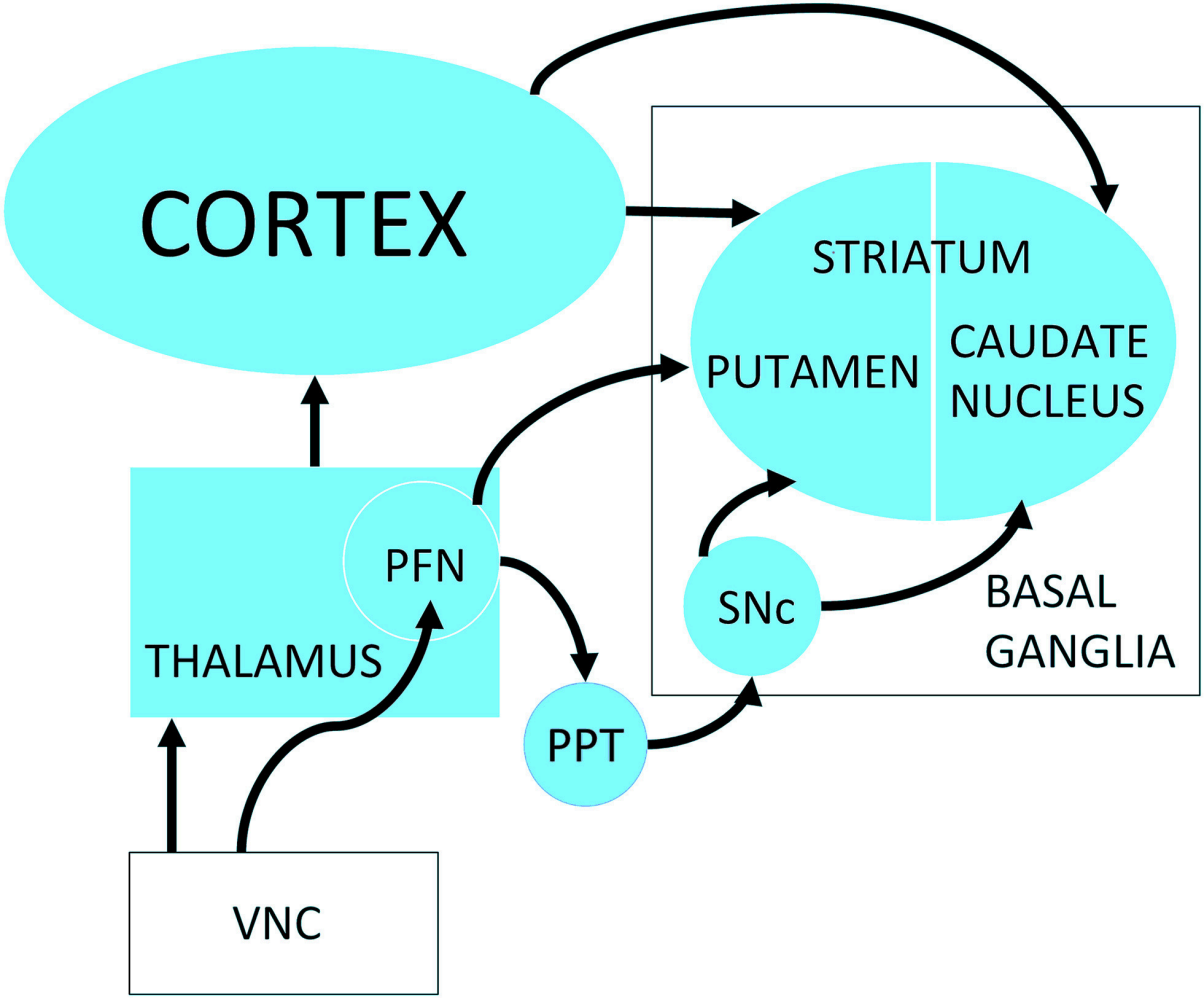
Possible neuronal pathways connecting the vestibular nucleus complex to the striatum. PFN, Parafascicular nucleus; PPT, pedunculopontine tegmental nucleus; SNc, Substantia nigra pars compacta; VNC, vestibular nucleus complex. Reproduced from Stiles et al. (59) with permission from the publisher.

The basal ganglia are a group of nuclei in the midbrain that are responsible for the coordination of movement as well as reinforcement learning. They comprise the dorsal striatum [the caudate nucleus and the putamen; see Figure 1] as well as the ventral striatum (the nucleus accumbens) and the globus pallidus [see (47) for a review]. There is evidence that the vestibular system may have a substantial influence over the basal ganglia, due to the need to integrate information about self-motion with plans to initiate voluntary movement, including voluntary eye movement [see (6, 22) for reviews].

Vestibular information was first proposed to be transmitted to the striatum via the motor cortex [e.g., (48)] or the hippocampus [e.g., (49)]. However, Muskens (50, 51) suggested that it may be transmitted via subcortical pathways. Potegal et al. (52) sought to test Muskens' hypothesis by lesioning the “vestibular cortical projection area” and recording from the caudate nucleus while electrically stimulating the vestibular nerve. If vestibular input to the caudate nucleus arose from the vestibular cortex and required it to be intact, then lesioning the latter should abolish vestibular responses in the caudate nucleus. However, they found no change in the evoked field potentials in the caudate nucleus with the vestibular cortex inactivated, suggesting the possibility of subcortical pathways between the vestibular nucleus complex and/or cerebellum and the dorsal striatum. Further field potential studies in both the caudate nucleus and putamen of the dorsal striatum demonstrated responses to electrical stimulation of the vestibular nerve [squirrel monkeys; (53)] or the lateral and medial vestibular nucleus [cats; (54)]. On the other hand, the results of single neuron studies were ambiguous. Segundo and Machne (55) reported that electrical stimulation of the vestibular labyrinth in cats resulted in an increase in the firing rate of single neurons in the putamen and the globus pallidus. By contrast, Matsunami and Cohen (56) found no change in the firing of single striatal neurons in the caudate nucleus of awake rhesus monkeys, in response to electrical stimulation of the contralateral vestibular nucleus complex, with the exception of when stimulation trains were used and the current intensity was high enough to produce movement of the limbs. More recently, Rancz et al. (46) reported that field potentials and multi-unit activity could be evoked in the striatum in rats in response to electrical stimulation of the superior vestibular nerve. They also confirmed this result using fMRI.

**Figure 2:**
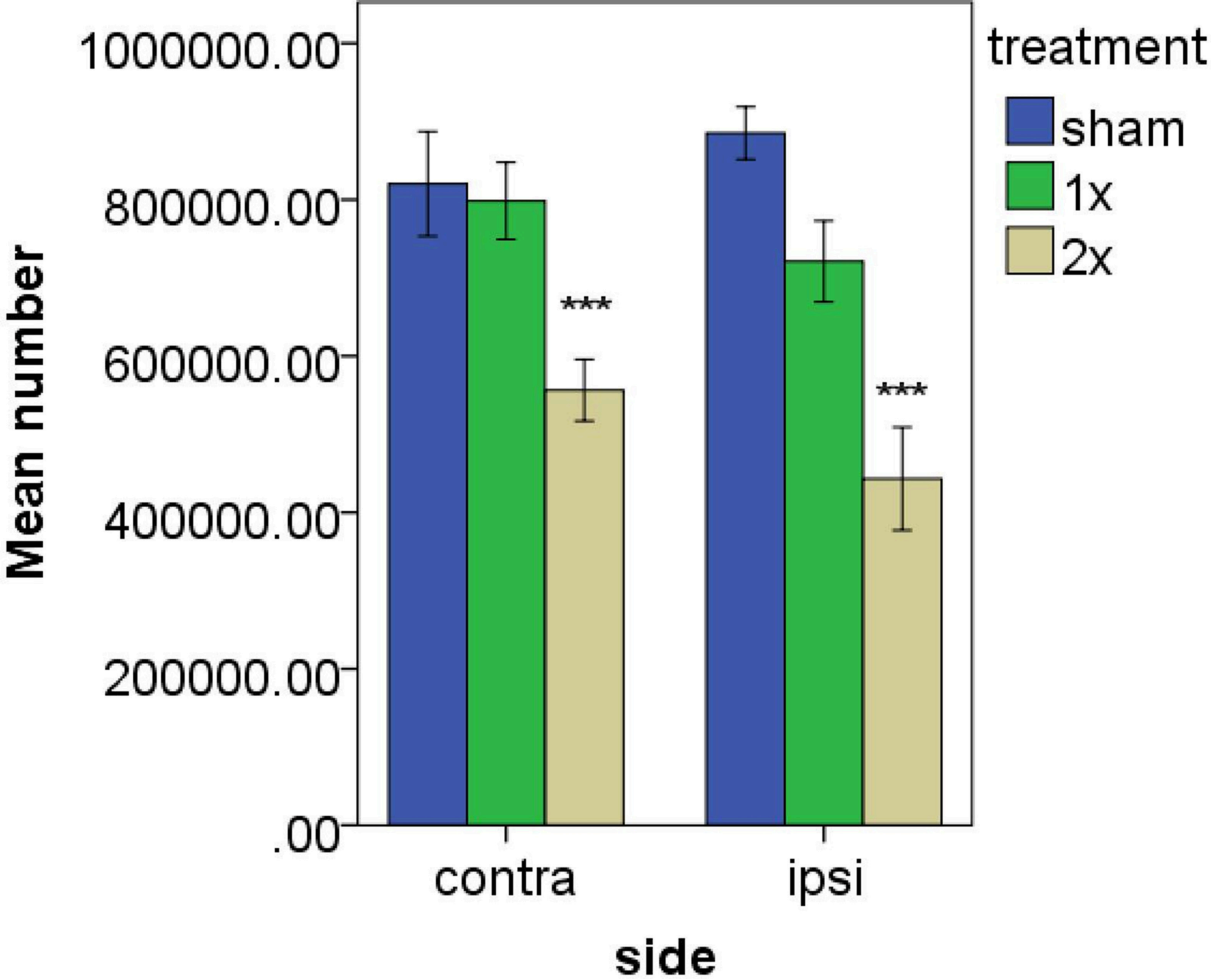
Estimated number of c-Fos positive cells in the striatum following vestibular stimulation. ^***^P ≤ 0.0001 for the comparison of the higher current with both the sham groups and the lower current group. From Stiles et al. (59) with permission.

**Figure 3:**
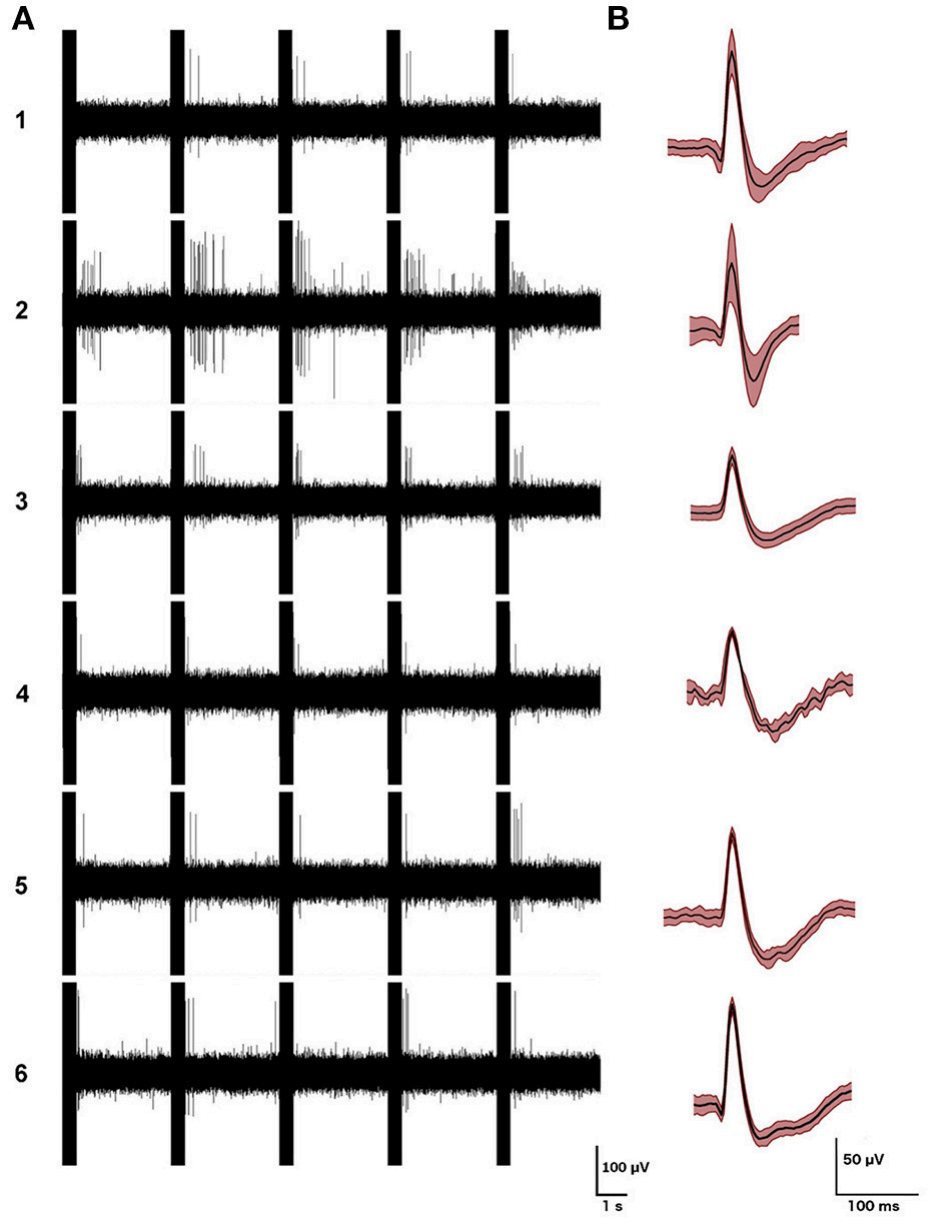
Examples of the firing patterns of the 6 single striatal neurons responding to electrical stimulation of the vestibular labyrinth in a phase-locked manner, **(A)** with examples of their action potential waveforms (averages of 200 action potentials **(B)**; mean ± SD in red). From Stiles et al. (59) with permission.

**Figure 4:**
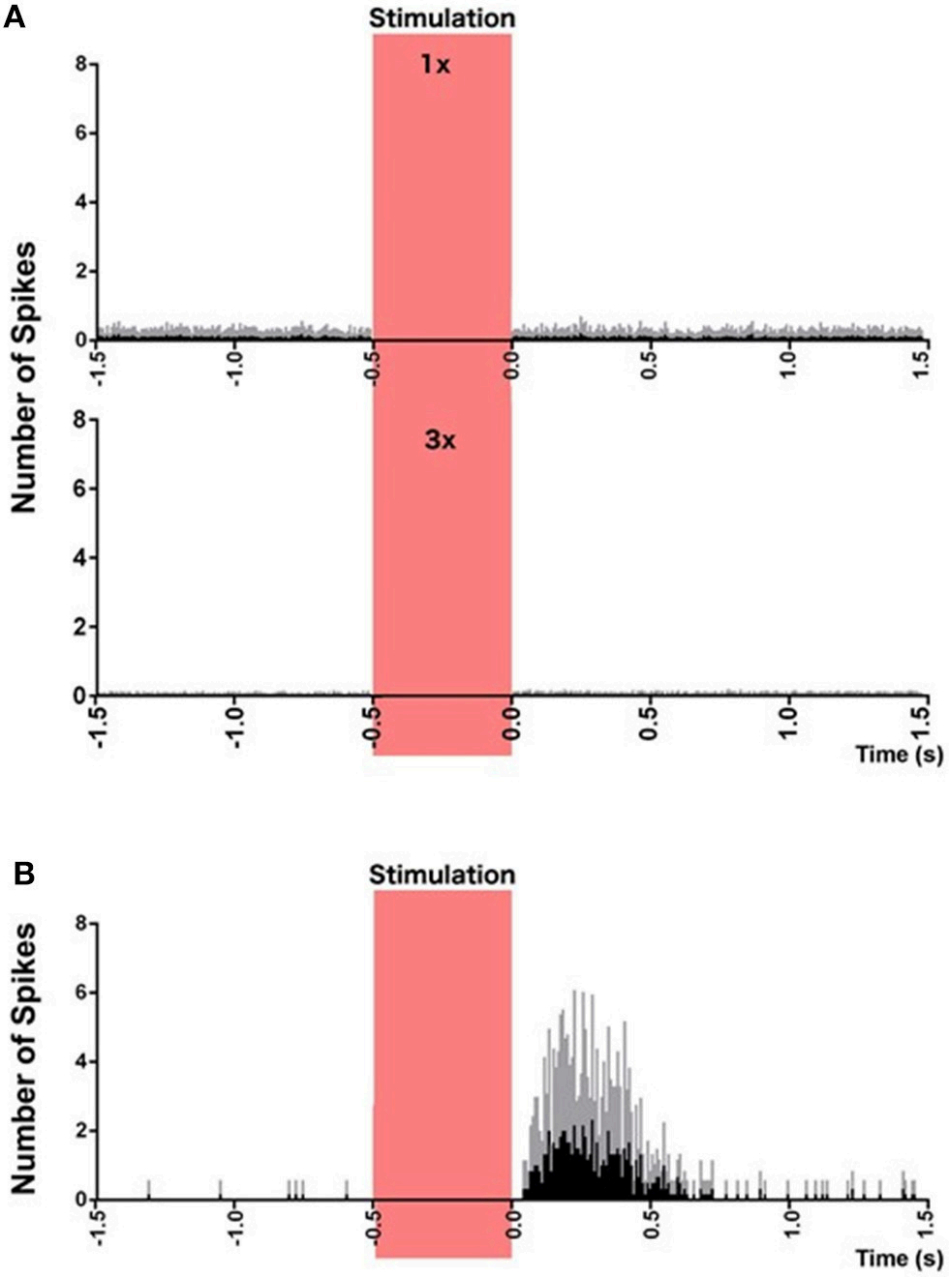
Peri-stimulus histograms of neuronal responses to electrical vestibular stimulation. **(A)** Combined histogram of firing of all non-responsive neurons at 1 × (top) and 2 × (bottom) the threshold of nystagmus. **(B)** Combined firing of all 6 responsive neurons, at 3 × the threshold of nystagmus, phase-locked to the stimulus. Red bar represents the stimulation period. Spikes from the stimulus artifact have been removed for clarity. Data are presented as mean (black bars) and standard deviation (gray bars). From Stiles et al. (59) with permission.

Striatal neurons have been demonstrated to fire in response to movement that is in phase with head velocity, suggesting the possibility of vestibular input from the vestibular nucleus complex or cerebellum (57, 58). Stiles et al. (59) have also recently reported that c-Fos expression, as a marker of cellular activation, and the firing rate of a circumscribed number of single striatal neurons, can be altered by electrical stimulation of the vestibular labyrinth in the anesthetized rat (see Figures 2–4). In related studies, they also demonstrated that such electrical stimulation can modulate the release of serine, threonine and taurine, as well altering DA metabolism (60).

Lai et al. (61) conducted neurotracer studies that suggested rapid pathways between the vestibular nucleus complex and/or cerebellum and the dorsolateral putamen, via the parafascicular nucleus of the thalamus (see Figure 1). On the basis of these results they suggested that there may be a disynaptic pathway from the vestibular nucleus complex and/or cerebellum to the striatum. Recently, Kim et al. (62) reported that polysynaptic field potentials could be evoked in the contralateral parafascicular nucleus following electrical stimulation of the horizontal semi-circular canal vestibular nerve in rats. Stiles et al. (59) observed some increases in the response of single striatal neurons to electrical stimulation of the vestibular labyrinth, with latencies of approximately 50 ms, which, under urethane anesthesia, could be consistent with a disynaptic pathway (63).

Many animal studies have attempted to understand the impact of the vestibular system on the basal ganglia, by lesioning the peripheral vestibular system or by using transgenic animals lacking vestibular function. There have been reports of vestibular loss affecting the expression of DA receptors in the striatum (64). Giardino et al. (64) reported that in young and old rats, unilateral peripheral vestibular lesions resulted in a bilateral increase in D_1_ DA receptors in the striatum, as well as an increase in D_2_ receptors. Bilateral vestibular loss, however, did not affect D_1_ receptor density in young rats while it reduced D_2_ receptors. By comparison, bilateral vestibular loss resulted in increased D_1_ and D_2_ receptors in the striatum in old rats. However, other studies have failed to find similar changes in the number of DA receptors (65). Stiles et al. (65) reported that D_2_ receptors were significantly higher in number in the right striatum than the left in both sham control and bilateral vestibular loss rats.

One of the most dramatic symptoms of bilateral vestibular loss in rodents is locomotor hyperactivity and circling behaviors (64, 65). Eugene et al. (66) studied circling behavior in the vestibular-deficient *KCNE1* mutant mouse and reported that it was associated with increased tyrosine hydroxylase expression, a marker for DA synthesis, in the striatum ipsilateral to the direction of circling, whether it was in a leftward or rightward direction. This increase in circling and locomotor activity observed in vestibular-deficient animals may suggest a change in striatal function resulting in a hyperkinetic disorder (67). The results from vestibular-deficient *ci2/ci2* rats are also consistent with this hypothesis: the specific DA D_2_ receptor antagonist, raclopride, caused a decrease in locomotor hyperactivity and circling behavior (68). However, Antoine et al. (69) found no change in DA receptors in a genetic mouse model of vestibular dysfunction in which the *Sk12a2* gene was knocked out in the inner ear, specifically disrupting vestibular function. They did, however, find a significant increase in the amount of phosphorylated extracellular signal-regulated kinase 1/2 (ERK1/2) and its downstream target, phosphorylated cyclic AMP response element binding protein (pCREB), in the nucleus accumbens in the ventral striatum. The lack of change in the non-phosphorylated ERK1/2 suggested an increase in activation of the ERK1/2 pathway, which is involved in learning and memory in the basal ganglia.

A different explanation of locomotor hyperactivity associated with vestibular loss, comes from a study by Pan et al. (70), who demonstrated a correlation between induced motor hyperactivity in rats and the increased labeling of orexin A neurons in the hypothalamus following bilateral vestibular loss. Orexin, or hypocretin, is a peptide secreted by small groups of neurons in the hypothalamus, which project to various brain regions such as the cortex, basal forebrain, dorsal raphe, pedunculopontine tegmental nucleus, tuberomammillary nuclei and locus coeruleus. Orexin regulates the sleep-wake cycle and low levels of it are the cause of narcolepsy. Interestingly, orexin neurons have also been shown to project to the vestibular nucleus complex (see (71) for a review). Pan et al. (70) found that the locomotor hyperactivity caused by bilateral vestibular loss was reduced by a type A orexin receptor antagonist. Nonetheless, the locomotor activity of the control rats was also reduced, suggesting a non-specific effect.

Other studies have found neurochemical changes in the striatum following bilateral vestibular loss in rats. Aitken et al. (72), using receptor autoradiography, reported that M_1_ muscarinic acetylcholine receptors, which mediate many of the excitatory effects of acetylcholine, decreased in density in the striatum and the hippocampus at 30 days (but not 7 days) following bilateral vestibular loss induced by intratympanic injection of sodium arsanilate. In a related study, Benoit et al. (73), using flow cytometry, demonstrated that the number of neurons expressing M_2_ acetylcholine receptors, which mediate many of the inhibitory effects of acetylcholine, underwent a significant increase at 30 days (but not 7 days) in the striatum and hippocampus following bilateral vestibular loss. Benoit et al. (74) have also reported that the number of striatal neurons expressing N-methyl-D-aspartate receptors exhibited a significant decrease at 7 days (but not 30 days) following bilateral vestibular loss.

Samoudi et al. (75) used hemiparkinsonian rats (the 6-hydroxy-dopamine model) to examine the effects of nGVS on motor symptoms and neurotransmitter release in the basal ganglia. They found that nGVS improved locomotor activity, measured by performance on a rotarod, and enhanced GABA release in the substantia nigra; however, DA release in the striatum was not significantly affected. The only other study to use microdialysis following high frequency stimulation of the vestibular labyrinth in rats, i.e., not GVS or nGVS, showed no significant effect on DA release in the striatum; however, there was evidence of a significant reduction in DA metabolism, as demonstrated by a reduced ratio between 3,4-dihydroxyphenylacetic acid and DA (60).

Taken together, the evidence from animal studies strongly suggests that vestibular input is transmitted to the basal ganglia, the striatum in particular. However, the nature of this input is complex and may be restricted to specific areas of the striatum. For example, the results of the available electrophysiological studies are complicated and difficult to reconcile with some of the other neurochemical data. Some of these apparent discrepancies are likely to be due to the anesthetic conditions under which the electrophysiological experiments were conducted. However, the behavioral evidence indicates that bilateral vestibular loss has major effects on motor activity in rodents.

### Human Studies

Bottini et al. (76), using PET scans, reported an increase in activity in the putamen (part of the dorsal striatum) following cold water caloric vestibular stimulation in healthy subjects [see (77) for a review]. Using GVS, Bense et al. (78) obtained similar results in the putamen. Other PET and fMRI studies in humans have reported increases in activity in the putamen and the caudate nucleus, following either cold caloric vestibular stimulation or galvanic vestibular stimulation (GVS) [(79–82); see (77) for a review]. An interesting recent result is that people with persistent postural perceptual dizziness have been shown to exhibit a decrease in gray matter volume in the caudate nucleus (83).

Jansen et al. (84) investigated D_2/3_ receptors in patients with bilateral vestibular loss and found that they exhibited an approximately 40% decrease in the bilateral temporo-parieto-occipital cortex, as well as in the striatum and the right thalamus. The longer the disease duration, the greater was the loss of D_2/3_ receptors in the middle/superior temporal gyrus. Patients who suffered from oscillopsia exhibited reduced D_2/3_ receptor availability in the right medial temporal and medial superior temporal regions.

Overall, the available human data are consistent in supporting the notion that the basal ganglia receive vestibular input, and some recent studies suggest that the striatum may undergo changes in conditions such as persistent postural perceptual dizziness. Furthermore, vestibular loss appears to alter the expression of DA receptors in the human brain.

## Effects of Vestibular Stimulation on Parkinsonian Symptoms

Many studies have investigated the potential of vestibular stimulation to reduce the severity of Parkinsonian symptoms. Most of these studies have employed sub-threshold GVS with a Gaussian noise signal superimposed upon it—so-called “stochastic or noisy GVS (nGVS).” The principle behind its effects is known as “stochastic resonance”: that a sub-threshold sensory signal may be more effectively detected by the brain if a noise signal is superimposed upon it (see (85) for a review). Having said this, the effects of nGVS upon the brain are poorly understood and there is a sense in which the application of it to neurological disorders has preceded a scientific understanding of its neural effects.

One of the first studies was by Yamamoto et al. (7), who investigated the effects of 24 h of nGVS on 7 patients with multi-system atrophy and 12 patients with L-DOPA-responsive PD or L-DOPA-unresponsive PD. They reported that nGVS appeared to increase the speed of bradykinesic rest-to-active transitions, indicated by measurements of trunk activity in the PD patients. They also found that the stimulation decreased reaction time on a continuous performance task without any increase in omission or commission error rates, suggesting that the PD patients exhibited better motor execution during cognitive tasks.

Pal et al. (86) examined the effects of nGVS on postural sway in the medio-lateral and antero-posterior planes in 5 PD patients and 20 controls. The nGVS resulted in a small but significant decrease in sway, measured using center of pressure displacement over 26 s, in the eyes closed condition in the PD patients and controls with low intensity stimulation (0.1 mA).

Kataoka et al. (87) used normal GVS applied for 20 min to 5 PD patients. They reported that 3 out of 5 patients diagnosed with PD including postural instability and/or abnormal axial posture, exhibited a reduction in postural instability following the GVS stimulus. This was measured using the anterior and lateral bending angles (captured using 2 digital video cameras) while the patients were standing with their feet 10 cm apart, and their eyes open. Okada et al. (88) also employed normal GVS to study anterior bending posture in 7 patients with PD. They measured the patients' anterior bending angles while they stood with their eyes open or closed. They found that the GVS significantly reduced the bending angles in both conditions compared to the sham control condition. However, the degree of change in the bending angle did not significantly correlate with the Unified Parkinson's Disease Rating Scale motor score, or the disease duration or the anterior bending angles before the GVS was applied.

Lee et al. (89) examined the effects of nGVS on tracking behavior in PD patients. They studied 12 PD patients with mild to moderate symptoms while they were off medication and asked them to perform a sinusoidal visuomotor tracking task, using a joystick. They found that the nGVS significantly increased the signal-to-noise ratio in the tracking task, enhancing the patients' ability to perform the task. The authors speculated that this effect may have been due to enhanced activity in the cingulate cortex.

Samoudi et al. (90) studied the effects of nGVS on motor symptoms in 10 PD patients who were either on or off L-DOPA. Following a backward perturbation, nGVS significantly improved balance corrections and reduced the response time, measured using a force plate and dynamic perturbation test. In the static posturography conditions, the nGVS significantly reduced the total sway with eyes closed when the patients were off L-DOPA. However, the nGVS increased nausea following L-DOPA administration in 2 subjects.

In the most recent study involving normal GVS, Koshnam et al. (91) examined its effects on motor symptoms in 11 PD patients while on medication. They employed both a timed up and go task as well as a finger tapping task and quantified the behavior using accelerometers and video cameras. They found that GVS significantly improved the coefficient of variation in step duration, the tapping score, and the duration of manual motor blocks.

To date, the studies of the effects of nGVS and GVS on PD have yielded fascinating data, which suggest the promise of potential novel therapies for the motor and non-motor symptoms of PD. However, it is important to keep in mind, at this early stage of investigation, the limitations of these studies. Most of them involve small sample sizes and when they included controls, the sample sizes were sometimes unequal [e.g., (86)]. Even when the patients served as their own controls in before and after studies, the issue of small sample sizes is important. No study conducted to date meets the standards of a randomized controlled clinical trial (RCT), in which PD patients would be randomly allocated to nGVS and sham nGVS groups, for example. Such a study would probably require double-blind measurement of the dependent variables, where neither the subject nor the experimenter knows to which treatment group the subject belongs, and the sample sizes employed would need to be based on statistical power calculations. In this kind of study, it would be important to separate the vestibular contributions to balance from other contributions such as proprioceptive inputs, and to measure vestibular function more broadly, including the VOR and VEMPs. Finally, it would be ideal to include non-motor as well as motor symptoms of PD, in order to determine the effects of nGVS and GVS on cognitive function and depression.

Wilkinson et al. (8) employed caloric vestibular stimulation to examine the effects of vestibular activation in a single case study of PD. Compared to baseline and the sham condition, they observed improvements in the scores for the EQ5D (a standardized instrument for quantifying general health status), Unified Parkinson's Disease Rating Scale, the Schwab and England Activities of Daily Living Scale (a scale which measures the ability of PD patients to function independently), 2 min walk, timed up and go, non-motor symptom assessment scale for PD, Montreal cognitive assessment scale, Hospital depression scale and Epworth sleepiness scale. These changes exceeded the minimal clinically important difference thresholds for these measures. This study, although based on a single patient, suggests the possibility that noisy vestibular stimulation may not be necessary in order to achieve clinical improvement with vestibular stimulation.

The effects of nGVS have also been studied in patients with vestibular dysfunction. Iwasaki et al. (92) studied 11 patients with bilateral vestibular loss and compared them to 21 healthy controls. Using white noise GVS they measured balance in terms of the velocity, the envelopment area and the root mean square center of pressure. They reported that the nGVS improved all 3 measures in 76% of the control subjects and 91% of the bilateral vestibular loss patients. They concluded that their study constitutes Class IV clinical evidence for the efficacy of nGVS in improving postural stability in patients with bilateral vestibular loss. Schniepp et al. (93) measured vestibulo-spinal reflex thresholds in 12 patients with complete bilateral vestibular loss and 10 with some residual function. They used a 1 Hz sinusoidal GVS to determine individual vestibulo-spinal reflex thresholds and then used nGVS. None of the patients with complete bilateral vestibular loss exhibited vestibulo-spinal reflex responses, as expected. However, they found that the delivery of weak nGVS improved the detection of subthreshold vestibular stimuli and reduced the threshold in 90% of the patients with residual vestibular function.

Some studies have also investigated the effects of nGVS in subjects without PD or any other neurological disorder. Goel et al. (94) delivered nGVS in the 0–30 Hz range to 45 subjects and measured the stability of the head, trunk and whole body. They reported that the stimulus delivered in the medio-lateral, anterior-posterior and combined directions significantly enhanced balance performance, measured using a force plate with motion sensors placed on the head and trunk. Pan et al. (95) examined the effects of 24 h of nGVS on wrist activity in 14 hospitalized patients, 10 with akinesia and 4 with ataxia. They found evidence from the power-law exponent that nGVS resulted in significantly reduced akinesia.

The only study to date, to investigate the electrophysiological effects of nGVS in humans, was by Kim et al. (96), who examined its effects on EEG. They measured theta (4–7.5 Hz), low alpha (8–10 Hz), high alpha (10.5–12 Hz), beta (13–30 Hz) and gamma (31–50 Hz) EEG bands in 10 neurologically-intact subjects. They found that the main effect of nGVS was to suppress the power of gamma EEG in lateral brain regions immediately following the stimulus, and that this was followed by a delayed increase in the power of beta and gamma EEG in frontal regions of the brain. The authors suggested that nGVS modulates the synchrony of multiple EEG oscillations. They speculated that the 1/*f* power density of the nGVS stimulus that they used may recruit more global neuronal networks at slower oscillations, which then affect higher frequency oscillations in networks of GABAergic interneurons, thus modulating many frequency bands (97).

A related question is whether vestibular stimulation through specific forms of vestibular rehabilitation, could be effective in the treatment of PD? Acarer et al. (98) studied the effects of vestibular rehabilitation in 29 PD patients and compared them to 11 control PD patients. Following 8 weeks of customized vestibular rehabilitation, they observed a significant improvement in scores in the Activities-Specific Balance (ABC) Confidence Scale (a scale measuring confidence in mobility), the Berg Balance Scale (which quantifies balance under different conditions such as standing up from a sitting position, standing on one foot etc.) and the Dynamic Gait Index (a measure of balance, gait and risk of falling). These results are consistent with those of Wilkinson et al. (8) and suggest that vestibular stimulation other than GVS or nGVS, may be useful in treating PD.

Taken together, the studies conducted in humans so far suggest that nGVS, and even normal GVS, may reduce postural instability and deficits in visual-motor control in patients with PD. There is also a suggestion that there may be some benefit to the non-motor symptoms of PD, although few studies have investigated this possibility so far. The fact that normal GVS, caloric vestibular stimulation and even vestibular rehabilitation on its own, may reduce some symptoms of PD, naturally raises the question of whether the stochastic property of nGVS is even necessary, or whether it is vestibular stimulation itself that is the key factor in any improvement. Future studies should compare these interventions under the same conditions in order to answer this question.

## Conclusions and Future Experiments

Although there are still relatively few studies of VOR function in patients with PD, there is increasing evidence that VEMPs, in particular, are abnormal. The evidence for abnormalities in the subjective visual vertical is less convincing, and much of the data supporting deficits is related to whether the patients exhibit lateral trunk flexion or whether they are on L-DOPA. There is some evidence for alterations in activity in the medial temporal area and cingulate sulcus visual area regions of the brain in response to visual motion stimulation and for abnormalities in the integration of information from different sensory modalities in PD.

There is substantial evidence for Parkinsonian neuropathological changes in the vestibular nucleus complex, including Lewy bodies (39) as well as reduced non-phosphorylated neurofilament and increased lipofuscin (44). There is also evidence for a reduction in cholinergic input to the thalamus (40), which is very interesting in light of the evidence for a decrease in pedunculopontine tegmental nucleus connectivity in the PD brain (43). The pedunculopontine tegmental nucleus is a major source of cholinergic input, contains neurons that are vestibular-responsive (41), and which undergoes significant changes in the number of acetylcholine-containing neurons following bilateral vestibular loss (42). It is very likely that the pedunculopontine tegmental nucleus is involved in the interaction between the vestibular nucleus complex, the parafascicular nucleus, which is part of the thalamus, and their connections with the substantia nigra and striatum (see Figure 1). Yousif et al. (99) reported that deep brain stimulation of the pedunculopontine tegmental nucleus in PD patients increased sway when going from light to darkness and also reduced vestibular perceptual thresholds.

Many imaging studies in humans have demonstrated that vestibular stimulation alters activity in the striatum (76–82). The results of electrophysiological studies in animals are more complex, especially the single neuron recording studies, of which there appear to be only three. It does appear that field potential changes are easier to record in the striatum in response to electrical stimulation of the peripheral vestibular system, at least in anesthetized rats (46, 59). Nonetheless, taken together with neurotracer studies [e.g., (61)] and other evidence from microdialysis studies (60), there is evidence for connections between the vestibular nucleus complex and cerebellum and the striatum. Certainly, more studies are needed to elucidate these connections, using selective electrical stimulation of the vestibular labyrinth and both neuronal recording and neurotransmitter microdialysis in the striatum (100, 101).

There is evidence that GVS and nGVS can reduce the severity of some PD symptoms (7, 86–91) and there is a case report that even caloric vestibular stimulation may have similar effects (8). However, more systematic studies are needed before the clinical effects of vestibular stimulation on PD become clear. Kim et al. (96) have provided fascinating data to suggest that nGVS modulates EEG activity in many frequency bands, and perhaps one of the most pressing needs in this area is the systematic investigation of the effects of nGVS on electrophysiological activity and neurotransmitter release in normal animals and also in animals exhibiting experimental Parkinsonian symptoms. These studies will elucidate the mechanism of action of nGVS in PD so that, if it is effective as an adjunctive treatment, its application can be optimized.

Finally, why is it that vestibular stimulation, in the form of nGVS, caloric vestibular stimulation or even natural vestibular stimulation, might exert beneficial effects on brain function in conditions such as PD? The answer to this question is elusive at present. However, it is conceivable that, due the evolutionary age of the vestibular system, and the otoliths in particular, their importance in detecting gravitational vertical and the widespread transmission of vestibular information across many brain regions, including many cortical areas, vestibular stimulation has some kind of “re-setting” effect on electrophysiological rhythms in the brain, which interferes with pathophysiological activity and promotes normal function (96). The precise details of how this happens and exactly what it entails will have to await further studies in animals and humans.

## Author Contributions

PS conceived and wrote the paper.

### Conflict of Interest Statement

The author declares that the research was conducted in the absence of any commercial or financial relationships that could be construed as a potential conflict of interest.

## Abbreviations

PD: Parkinson's Disease
GVS: galvanic vestibular stimulation
nGVS: noisy galvanic vestibular stimulation
DA: dopamine
GABA: γ-aminobutyric acid
L-DOPA, L-3: 4-dihydroxyphenylalanine
VORs: vestibulo-ocular reflexes
cVEMPs: cervical vestibular-evoked myogenic potentials
oVEMPs: ocular vestibular-evoked myogenic potentials
mVEMPs: masseter vestibular-evoked myogenic potentials
fMRI: functional magnetic resonance imaging
PET: positron emission tomography.
